# *In vitro* and molecular chemosensitivity in human cholangiocarcinoma tissues

**DOI:** 10.1371/journal.pone.0222140

**Published:** 2019-09-10

**Authors:** Manida Suksawat, Poramate Klanrit, Jutarop Phetcharaburanin, Nisana Namwat, Narong Khuntikeo, Attapol Titapun, Apiwat Jarearnrat, Prakasit Sa-ngiamwibool, Anchalee Techasen, Watcharin Loilome

**Affiliations:** 1 Department of Biochemistry, Faculty of Medicine, Khon Kaen University, Khon Kaen, Thailand; 2 Cholangiocarcinoma Research Institute, Khon Kaen University, Khon Kaen, Thailand; 3 Cholangiocarcinoma Screening and Care Program (CASCAP), Khon Kaen University, Khon Kaen, Thailand; 4 Department of Surgery, Faculty of Medicine, Khon Kaen University, Khon Kaen, Thailand; 5 Department of Pathology, Faculty of Medicine, Khon Kaen University, Khon Kaen, Thailand; 6 Faculty of Associated Medical Sciences, Khon Kaen University, Khon Kaen, Thailand; Texas A&M University, UNITED STATES

## Abstract

Adjuvant chemotherapy is required for cholangiocarcinoma (CCA) patients after surgical treatment. Gemcitabine and gemcitabine plus cisplatin are considered the appropriate regimen; however, the response spectrum to chemotherapy differs between patients. Thus, the present study aims to evaluate the response pattern of individual CCA patients by using an *in vitro* method, histoculture drug response assay (HDRA), to predict the chemosensitivity of individual patients in a prospective study. Moreover, we also investigate the expression of gemcitabine and cisplatin sensitivity factors in CCA tissues in the same cases. Based on the dose response curve, 1000 and 1500 μg/ml of gemcitabine were used as the testing concentrations. For cisplatin, concentrations of 20 and 25 μg/ml were selected for testing and for the combination regimen, 1000 μg/ml of gemcitabine and 20 μg/ml of cisplatin were chosen. The median %IR of each drug was measured as the cut-off to categorize the response pattern into response and non-response groups. In addition, we compared the effectiveness of the chemotherapy regimens between gemcitabine alone and gemcitabine plus cisplatin. The %IR of the combination of gemcitabine and cisplatin was significantly higher than gemcitabine alone. The relationship between the expression level of gemcitabine and cisplatin sensitive factors and the individual response pattern as well as clinicopathological data of CCA patients were analyzed. The results indicated that a low expression of the gemcitabine sensitive factor hENT-1 was significantly associated with the non-response group *in vitro* (*p* = 0.002). Moreover, the low expression of hENT-1 was also significantly associated with advanced stages CCA in the patients (*p* = 0.025). A low expression of MT and ERCC1 was significantly correlated with the response group in the *in vitro* experiments (*p* = 0.015 and *p* = 0.037 for MT and ERCC1, respectively). Therefore, HDRA may serve as an aid to selecting chemotherapy, and the expression of hNET-1, MT and ERCC1 may serve as biomarkers for predicting chemotherapy success.

## Introduction

Cholangiocarcinoma (CCA), an invasive bile duct cancer which originates from bile duct epithelium, is recognized as a major public health problem in northeastern Thailand, where it has the highest incidence worldwide. In this area it is associated with infection by the carcinogenic liver fluke (*Opisthorchis viverrini*, Ov), the major risk factor of CCA development [1]. The majority of CCA cases are clinically silent and most of the patients are diagnosed when the disease is at an advanced or metastatic stage with an extremely poor prognosis. Recurrence and progression of the tumor are very common after attempting curative surgery [2] and the survival rate of CCA patients still low [3]. Therefore, adjuvant chemotherapy is crucial in order to improve the survival. Various types of adjuvant chemotherapies are used in clinics [4], however, a standard chemotherapy for CCA patients has not been established. Based on the ABC trial, gemcitabine and gemcitabine plus cisplatin are commonly used in clinics for biliary tract cancer patients [5].

Pharmacogenomics, the variation in DNA occurring among individual patients, is considered to be a crucial factor for successful chemotherapy [6]. Therefore, a method that can accurately predict an individual’s response to chemotherapy for CCA is urgently required. Histoculture drug response assay (HDRA) is an *in vitro* chemosensitivity test which allows cancer cells to be cultured with native architecture, three-dimensional architecture, and also maintain the tissues organization [7, 8]. The clinical use of HDRA to predict chemosensitivity has been reported for various solid tumors, including breast, colorectal, lung and ovarian cancers [8–11]. Apart from the method, predictive biomarkers for chemotherapeutic response are also desired. The predictive biomarkers for gemcitabine sensitivity normally focus on proteins involved in the gemcitabine metabolic pathway, including deoxycytidine kinase (DCK), human equilibrative nucleoside transporter 1 (hENT-1) and ribonucleotide reductase subunit M1 (RRM1) [12, 13]. The correlation between the expression of all of these proteins and clinical outcome has been reported in various cancers [14–16]. The predictive biomarker for cisplatin sensitivity, metallothionein (MT) and the Excision repair cross complementation group 1 (ERCC1) have also been reported to be directly associated with the cisplatin response [17, 18].

Therefore, in the present study, HDRA was introduced to evaluate the sensitivity of chemotherapeutic agents including gemcitabine, cisplatin and gemcitabine plus cisplatin in the resected tumor tissues of individual CCA patients in a prospective study. Additionally, the expression of DCK, hENT-1, RRM1, MT and ERCC1 were investigated in human CCA tissues in the same cases. Then the relationship between the expression of candidate predictive markers and the individual dug response patterns was investigated.

## Patients and methods

### Patients and sample collection

Fresh surgically resected CCA tissues were obtained from patients who were diagnosed with CCA and had undergone surgery at Srinagarind Hospital, Khon Kaen University. The protocols for specimen collection and for the present study were approved by the Ethics Committee for Human Research, Khon Kaen University (HE571283 and HE601149, respectively). The specimens were collected during January 2017 until May 2019. The clinicopathological data of each patient were provided by the Cholangiocarcinoma Research Institute (CARI), Khon Kaen University and the Cholangiocarcinoma Screening and Care Program (CASCAP), Khon Kaen University.

The CCA tissues were divided into three parts. The first part was kept in 4°C Hank’s balanced salt solution (HBSS) with ciproflaxin, cefazolin, amphotericin B for submission into HDRA. The second part was fixed with 10% formalin and embedded in paraffin for immunohistochemical staining. The last part was stored at -80°C for further use.

### Histoculture drug response assay (HDRA)

**Fig 1:**
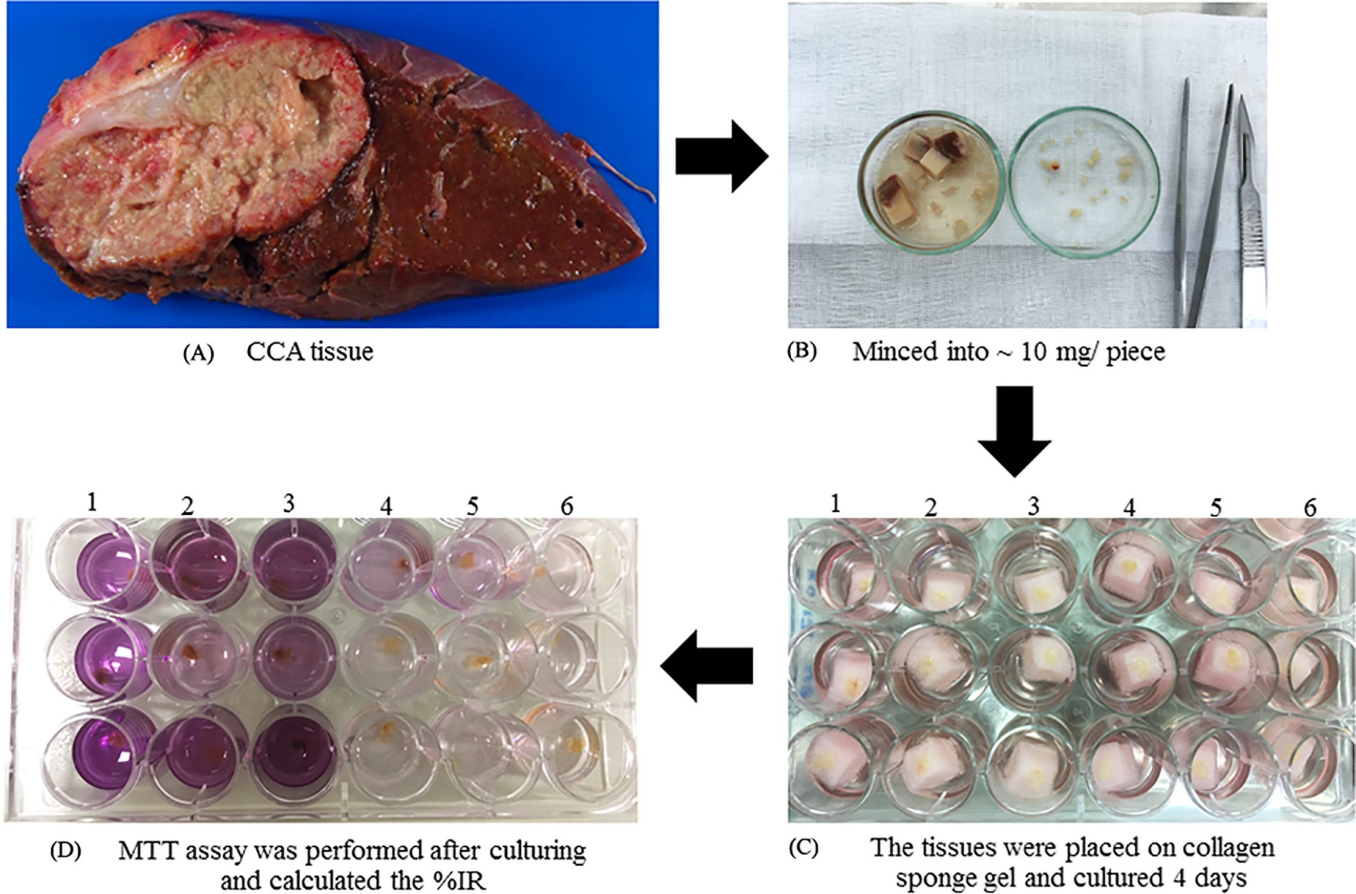
The protocol of HDRA method. (A) The fresh tumor tissue of CCA patients after surgery. (B) The tumor tissues were minced into small pieces and weight ~10 mg/piece. (C) The small piece of tumor tissues was placed on sponge gel and culture for 4 days in conditioned media without and with designing drug concentrations, respectively (D) After culturing, the tissues were performed MTT assay and calculated %IR in each condition. Conditions including 1 = control, 2 = 1000 μg/ml gemcitabine, 3 = 1500 μg/ml gemcitabine, 4 = 20 μg/ml cisplatin, 5 = 25 μg/ml cisplatin and 6 = 1000 μg/ml gemcitabine + 20 μg/ml cisplatin.

HDRA was performed according to the method previously described with minor adaptations. The tumor tissues were aseptically minced into ~ 10 mg pieces and placed on a collagen sponge gel (Ethicon, Inc. Summerville, United State) in a 24-well plate and cultured for 4 days in RPMI1640 medium (Thermo Fisher Scientific, Waltham, USA.) contained designed concentration of gemcitabine, cisplatin (Fresenius SE & Co. KGaA, Bad Homburg, Germany) and gemcitabine plus cisplatin, respectively. The culture media was supplemented with 20% fetal calf serum (Thermo Fisher Scientific, Waltham, USA.) penicillin (100 units/ml) and streptomycin (100 mg/ml) (Life Technologies, Inc., Carlsbad, USA.) and the assay was culture at 37°C in a 5% CO_2_ atmosphere. Each condition was tested in three culture wells. After culturing, 100 μl of HBSS (Thermo Fisher Scientific, Waltham, USA.) with 0.1 mg/ml collagenase type I (Thermo Fisher Scientific, Waltham, USA.) and 100 μl of MTT (Sigma Aldrich, St. Louis, USA.) solution were added into each well and the plates were incubated at 37°C for another 4 h. Next, the formazan crystals were extracted from the tumor tissues by dimethyl sulfoxide (DMSO) (Sigma Aldrich, St. Louis, MO, USA) and the absorbance of each well was measured by microplate reader at 540 nm (Tecan Sunrise, Switzerland). The briefly protocol was shown in Fig 1. Finally, the efficacy of the chemotherapeutic agents in each well was calculated as the % inhibition rate (%IR) as follows: %IR = 1-(T/C) x100, where T is the mean absorbance of the treated tumor/weight, and C is the mean absorbance of the control tumor/weight.

### Antibodies

Antibody to DCK (1:250, rabbit polyclonal directed against with human, mouse, rat) was purchased from GeneTex (Irvine, United State). Antibody to ERCC1 (1:100, mouse monoclonal directed against with human, rat) was purchased from Thermo Fisher Scientific (Waltham, USA). Antibody to hNET-1 (1:50, rabbit polyclonal directed against with human, mouse) was purchased from abnova (Taipei, Taiwan). Antibodies to RRM1 (1:250 rabbit monoclonal directed against with human, mouse, rat) and metallothinein (1: 50, mouse monoclonal directed against with human, mouse, rat, rabbit, dog) were obtained from abcam (Cambridge, UK)

### Immunohistochemistry

For the IHC staining, we followed the previous standard protocol for immunoperoxidase staining [19]. The sections of human CCA tissues were deparaffinized and rehydrated through xylene and increasing series of aqueous ethanol solutions. Then antigen retrieval was performed in sodium citrate plus Tween 20 buffer for 10 min using a microwave. Then endogenous peroxidase activity was blocked with 0.3% hydrogen peroxide in phosphate-buffered saline (PBS) for 30 min. In addition, 10% skim milk in PBS was used to block non-specific binding for 1 h. The tissue sections were then incubated with the primary antibody against the designated target proteins overnight at 4°C. Further, the tissue sections were washed in 0.1% (v/v) Tween-20 in PBS for 5 min (three times) followed by PBS for 5 min (once) and incubated with peroxidase-conjugated Envision^™^ secondary antibody (DAKO, Denmark, K4003) for 1 h at room temperature. The slides were then washed with 0.1% (v/v) Tween-20 in PBS for 5 min (three times) followed by PBS for 5 min (once). The 3,3'-diaminobenzidine (DAB) solution was used to develop color in the sections for 5 min. Then, the sections were counterstained with Mayer’s hematoxylin. Finally, the sections were dehydrated in a series of ethanol solutions of increasing concentration followed by xylene and then mounted.

An immunohistochemical scoring system for human CCA tissues was used for quantitation of the results. Scoring depended on the intensity and frequency of staining in the tumor area. The intensity of staining was classified into three groups: 1+, weak staining; 2+, moderate staining and 3+, strong staining. The intensity of staining was also defined in four groups: 0% = negative; 1+, 1–25%; 2+, 26–50%; and 3+,>50%. The staining score was calculated by multiplying frequency and intensity in each case, and the median score for all cases was calculated. The results were classified into two groups: in the low expression group the score was less than the median, in the high expression group the score was equal to or greater than the median.

### Statistical analysis

Statistical analysis was performed using SPSS software version 17 (IBM Corporation, NY). The comparison of effectiveness of gemcitabine and gemcitabine plus cisplatin was determined by paired t-test. The associations between the expression of proteins in human CCA tissue and clinico-pathological factors, as well as the in vitro method, were determined by Fisher’s exact test. *P*<0.05 was considered statistically significant.

## Results

### Patient characteristics

The characteristics of all CCA patients from whom tumor tissues were tested with gemcitabine in HDRA and that underwent IHC staining are summarized in Table 1. The characteristics of the patients, from whom tumor tissues were tested with cisplatin and that underwent IHC staining are shown in Table 2. In both groups, males comprised the majority of patients. The median age was 63. In addition, the tumors of both groups occurred intrahepatically with non-papillary type histology. Margin free status was reached for most of population and most patients had CCA at stages III and IV at the time of diagnosis.

**Table 1:** The characteristics of all CCA patients for whom the tumor tissues were tested with gemcitabine using HDRA.

Variables	Number (n)
Sex	
Male	19
Female	15
Age (year)	
Less than 63	16
63 or greater	18
Tumor site	
Intrahepatic	22
Extrahepatic	12
Histology	
Papillary	13
Non-papillary	21
Marginal status	
Free margin	18
Not free margin	12
Not applicable	4
Primary tumor (T)	
Is, I, II	10
III, IV	22
Not applicable	2
Reginal lymph node (N) metastasis	
No	15
Yes	10
Not applicable	9
Distance metastasis	
No	17
Yes	6
Not applicable	11
TNM stage	
I,II	6
III,IV	25
Stage unknown	3

**Table 2:** The characteristics of all CCA patients for whom the tumor was tested with cisplatin using HDRA.

Variables	Number (n)
Sex	
Male	22
Female	11
Age (year)	
Less than 63	15
63 or greater	18
Tumor site	
Intrahepatic	22
Extrahepatic	11
Histology	
Papillary	9
Non-papillary	24
Marginal status	
Free margin	18
Not free margin	11
Not applicable	4
Primary tumor (T)	
Is, I, II	13
III, IV	18
Not applicable	2
Reginal lymph node (N) metastasis	
No	14
Yes	10
Not applicable	9
Distance metastasis (M)	
No	15
Yes	5
Not applicable	13
TNM stage	
I,II	7
III,IV	23
Stage unknown	3

### Dose response analysis

**Fig 2:**
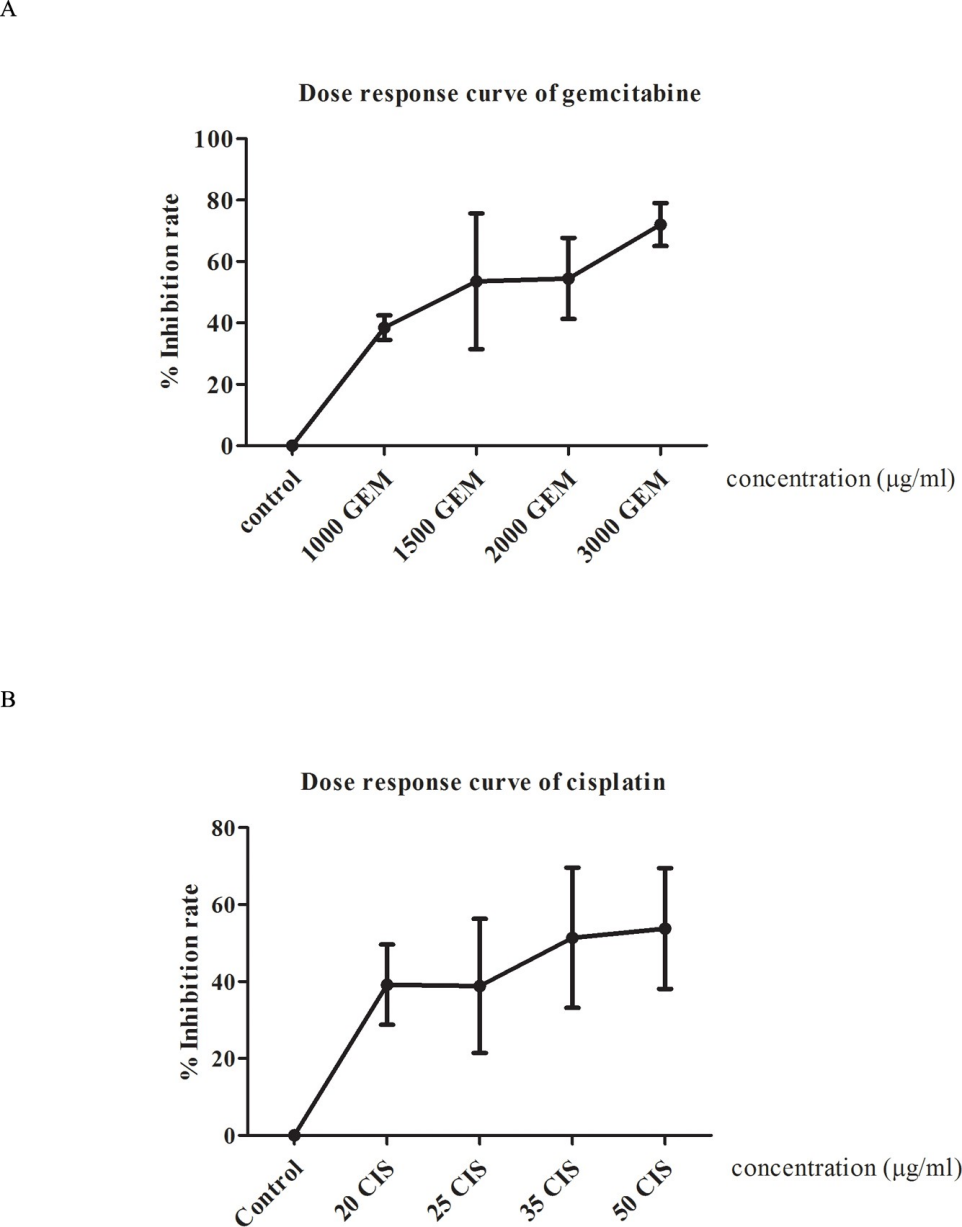
Dose response analysis of gemcitabine and cisplatin. X axis represents each concentration of each drug. Y axis represents %IR of each concentration in each drug. (A) Represent dose response curve of gemcitabine in concentration 1000, 1500, 2000 and 3000 μg/ml were tested to observed %IR. (B) Elucidate dose response curve of cisplatin in concentration 20, 25, 35, 50 μg/ml of cisplatin were also tested to observed %IR.

The dose response analysis in HDRA was firstly performed in order to evaluate the concentration and cut-off used to classify the tumor tissues of patients into response and non-response groups for each drug. Various concentrations of gemcitabine and cisplatin were tested with 5 cases of CCA tissues per drug. The dose response curve and the %IR of gemcitabine and cisplatin are shown in Fig 2. Concentrations of 1000 and 1500 μg/ml of gemcitabine were selected as test concentrations with cut-offs of 38.46% and 43.28%, respectively, while concentrations of 20 and 25 μg/ml of cisplatin were chosen as test concentrations with cut-offs of 39.05% and 32.65%, respectively. Moreover, we also evaluated the pattern of the combination of gemcitabine and cisplatin. The lowest concentration of each drug, 1000 μg/ml of gemcitabine and 20 μg/ml of cisplatin, was selected as the test concentration.

### Ability of chemotherapeutic agents to inhibit growth of CCA tissues

After the dose response assay, the selected concentrations of gemcitabine, cisplatin and gemcitabine plus cisplatin were used to evaluate the individual response pattern of CCA patients to the chemotherapies. A total of 34 cases of tumor tissues were tested with gemcitabine, and 33 cases were tested with cisplatin. In addition, 24 cases of CCA tissues were tested with gemcitabine and gemcitabine plus cisplatin in HDRA. The HDRA results indicate that the most of tumor tissues of CCA patients seem to be non-responsive to gemcitabine at both concentrations, as is also the case for 20 μg/ml cisplatin. However, 25 μg/ml of cisplatin increases the number of responsive cases (Table 3).

**Table 3:** The ability of gemcitabine and cisplatin to inhibit tumor tissue growth.

Chemotherapies	Cases (n)
1000 μg/ml gemcitabine	
response	7
non-response	27
1500 μg/ml gemcitabine	
response	6
non-response	28
20 μg/ml cisplatin	
response	9
non-response	24
25 μg/ml cisplatin	
response	18
non-response	15

Based on the ABC trial, we further explored the response pattern of tumor tissues with the combination of gemcitabine and cisplatin and compared %IR of this condition with the gemcitabine treated group. The results indicated that the combination of gemcitabine and cisplatin inhibited tumor growth significantly more than gemcitabine alone (*p*<0.001) (Table 4). From this result we can conclude that the combination of gemcitabine and cisplatin shows better clinical efficiency than gemcitabine alone.

**Table 4:** Comparison the %IR between gemcitabine and gemcitabine plus cisplatin.

Regimens	Mean %IR	Paired Difference	
		95% CI (lower/upper)	*p*-value
Gemcitabine	-12.132	-69.718/-39.744	<0.001*
Gemcitabine plus cisplatin	42.599		

*P<0.005, statistically significant

### Expression of candidate predictive biomarkers in human CCA tissues

**Fig 3:**
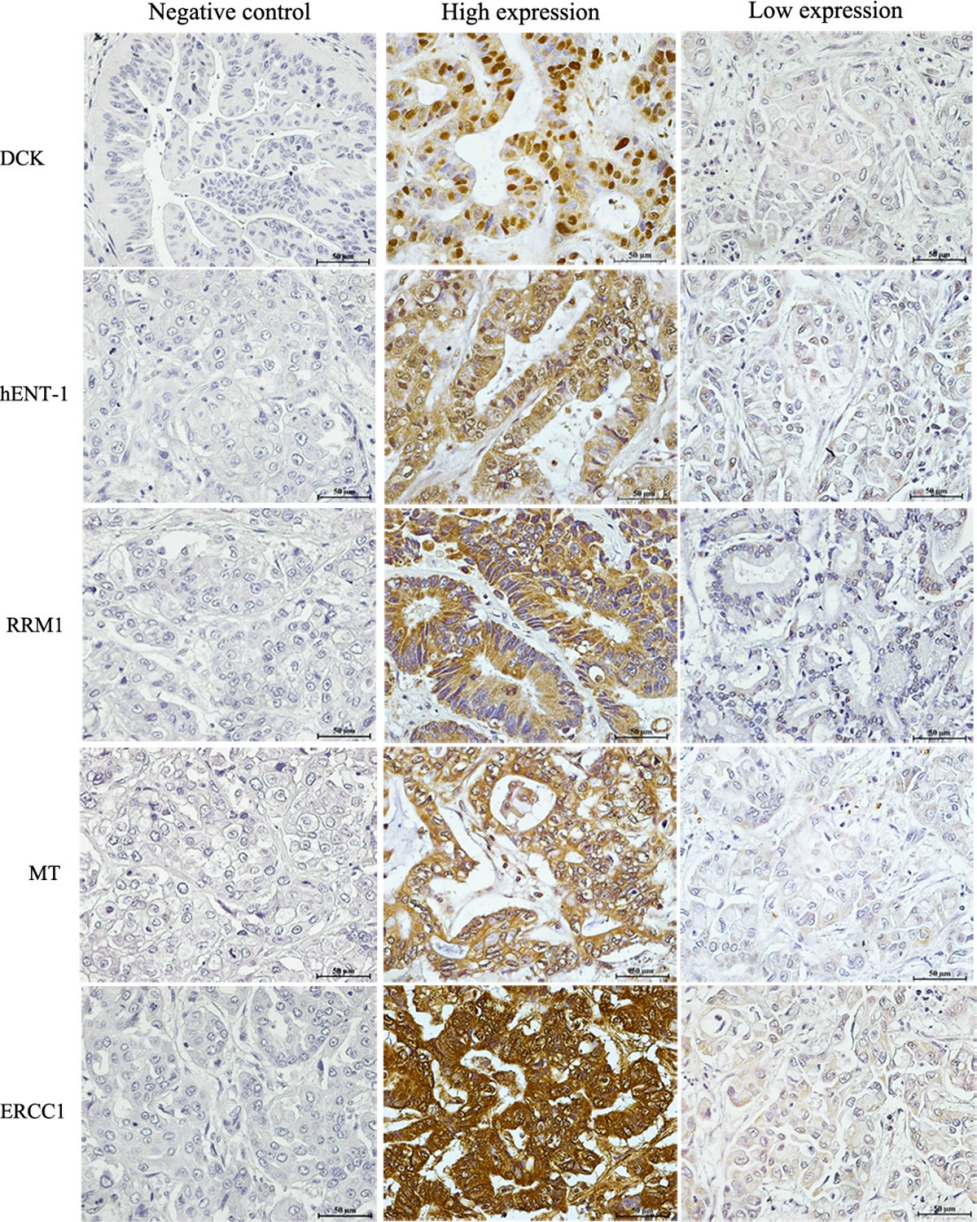
The expression level of gemcitabine and cisplatin candidate predictive biomarkers in human CCA tissue. The left panel represents negative control of each protein. The middle panel illustrates high expression of each protein and right panel represents low expression of each protein. Bar 50 μm (insert).

We further examined the expression of DCK, hENT-1, RRM1, MT and ERCC1 in CCA tissues in order to explore their capacity for the prediction of gemcitabine and cisplatin response. The representative expression of each protein is shown in Fig 3. Gemcitabine sensitive factors DCK, hENT-1 and RRM1 showed a high expression of 44.12%, 14.70% and 47.06%, respectively. While the expression of the MT and ERCC1 cisplatin sensitive factors showed a high expression of 48.48% and 51.52%, respectively.

### Correlation of predictive biomarkers with response pattern from HDRA and clinicopathological data of CCA patients

**Fig 4:**
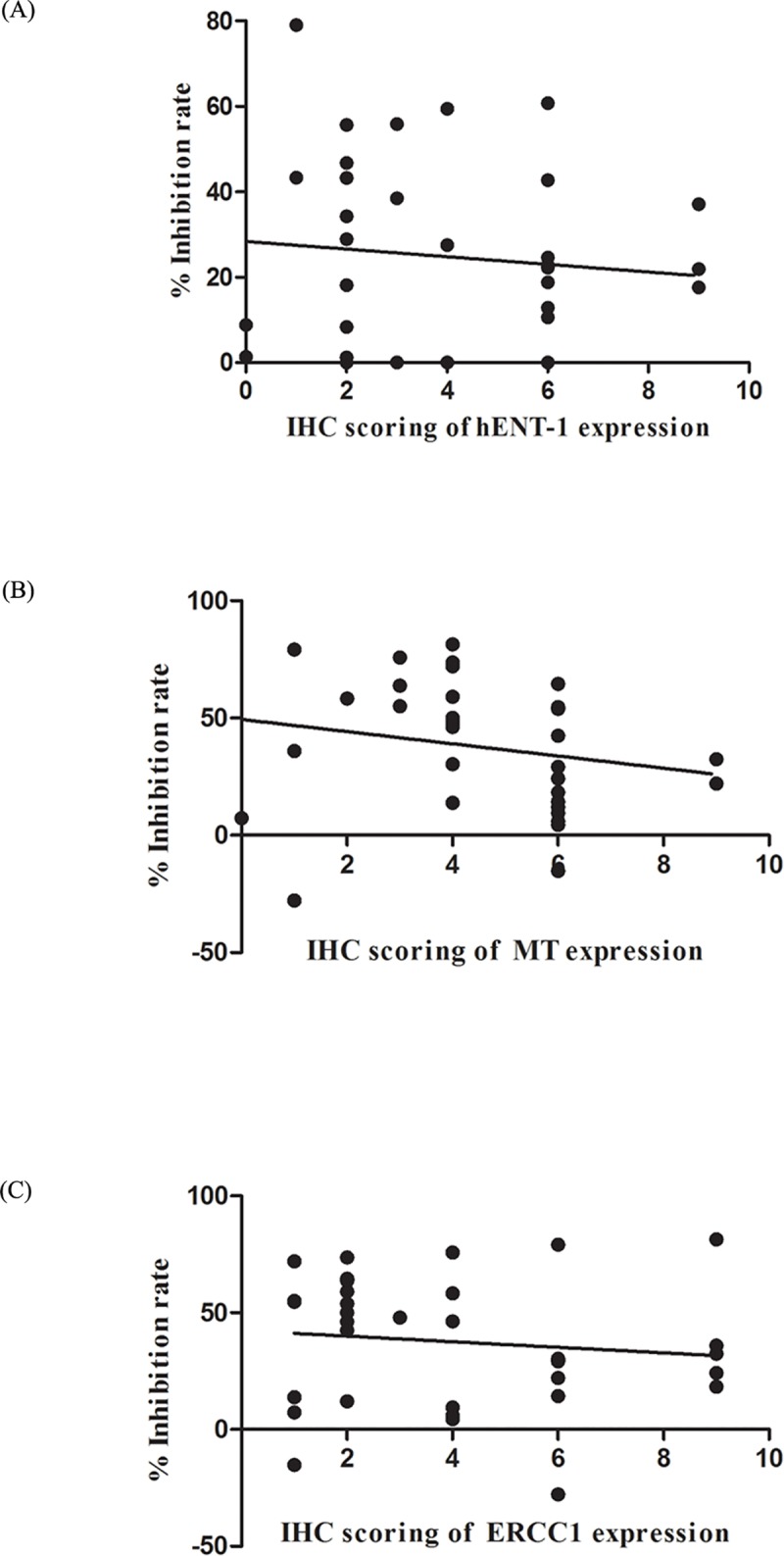
The correlation of expression of gemcitabine and cisplatin predictive biomarkers and %IR. X axis represents IHC scoring of hENT-1, MT and ERCC1 (A, B and C, respectively). Y axis represent %IR of 1500 μg/ml of gemcitabine and 25 μg/ml of cisplatin (A and B, C, respectively). (A) Elucidate the negative correlation of the expression of hENT-1 and %IR of 1500 μg/ml of gemcitabine. (B) Elucidate the negative correlation of the expression of MT and %IR of 25 μg/ml of cisplatin. (C) Illustrate the negative correlation of the expression of ERCC1 and %IR of 25 μg/ml of cisplatin.

We further analyzed the association between the candidate biomarker expression with the HDRA drug response pattern and the patient’s clinical data. The results of the relationship between the expression of the predictive biomarkers of gemcitabine with gemcitabine response from HDRA revealed that a low expression of hENT-1 was significantly associated with the tumor tissues non-responsive to gemcitabine at a concentration of 1500 μg/ml (*p* = 0.002). The negative correction of hENT-1 expression and %IR of 1500 μg of gemcitabine was shown in Fig 4A. Moreover, correlation of the expression level and the clinicopathological data of CCA patients shows that a low expression of hENT-1 was also significantly associated with advanced stage (III and IV) CCA patients (*p* = 0.025) (Table 5).

**Table 5:** Correlation of the expression of DCK, hENT-1 and RRM-1 with the drug response pattern using HDRA and the clinicopathological data of CCA patients.

Variables	N (33)	DCK			hENT-1			RRM1		
		Low	High	*p*	Low	High	*p*	Low	High	*p*
1000 μg/ml gemcitabine										
Non-response	27	15	12	1.000	24	3	0.268	16	11	0.241
Response	7	4	3		5	2		2	5	
1500 μg/ml gemcitabine										
Resistance	28	16	12	1.000	27	1	0.002^*^	14	14	0.660
Sensitive	6	3	3		2	4		4	2	
Sex										
Male	19	13	6	0.165	14	5	0.053	12	7	0.300
Female	15	6	9		15	0		6	9	
Age (year)										
Less than 63	16	7	9	0.300	14	2	1.000	9	7	0.744
63 or greater	18	12	6		15	3		9	9	
Tumor site										
Intrahepatic	22	14	8	0.288	18	4	0.635	9	13	0.080
Extrahepatic	12	5	7		11	1		9	3	
Histology										
Papillary	13	8	5	0.728	13	0	0.132	8	5	0.497
Non-papillary	21	11	10		16	5		10	11	
Marginal status										
Free margin	18	11	7	0.411	15	3	0.677	12	6	0.199
Not free margin	12	5	7		11	1		4	8	
Not applicable	4	3	1		3	1		2	2	
Primary tumor (T)										
Is, I, II	10	5	5	0.878	8	2	0.251	6	4	0.290
III, IV	22	13	9		20	2		10	12	
Not applicable	2	1	1		1	1		2	0	
Reginal lymph node (N) metastasis										
No	17	11	6	0.442	16	1	0.344	10	7	0.769
Yes	8	3	5		6	2		4	4	
Not applicable	9	5	4		7	2		4	5	
Distance metastasis (M)										
No	17	8	9	0.389	15	2	0.281	9	8	0.704
Yes	6	3	3		6	0		4	2	
Not applicable	11	8	3		8	3		5	6	
TNM stage										
I,II	8	5	3	0.581	6	2	0.025^*^	5	3	0.490
III,IV	25	13	12		23	2		12	13	
Stage unknown	1	1	0		0	1		1	0	

*P<0.005, statistically significant

The relationship of the expression of the predictive biomarkers for cisplatin and cisplatin response in HDRA indicated that a high expression of MT and ERCC1 was significantly associated with tumor tissues non-responsive to 25 μg/ml of cisplatin (*p =* 0.015 and *p* = 0.037, for MT and ERCC1 respectively) (Table 6). Moreover, the negative correlation of the expression of MT and ERCC1 with %IR of 25 μg/ml of cisplatin was shown in Fig 4B and 4C.

**Table 6:** Correlation of the expression of MT and ERCC1 with the drug response pattern using HDRA and the clinicopathological data of CCA patients.

Variables	N (33)	MT			ERCC1		
		Low	High	*p*	Low	High	*p*
20 μg/ml cisplatin							
Non-response	24	10	14	0.118	9	15	0.057
Response	9	7	2		7	2	
25 μg/ml cisplatin							
Resistance	15	4	11	0.015*	4	11	0.037*
Sensitive	18	13	5		12	6	
Sex							
Male	22	12	10	0.721	12	10	0.465
Female	11	5	6		4	7	
Age (year)							
Less than 63	15	8	7	1.000	6	9	0.491
63 or greater	18	9	9		10	8	
Tumor site							
Intrahepatic	22	11	11	1.000	11	11	1.000
Extrahepatic	11	5	6		6	5	
Histology							
Papillary	10	7	3	0.259	5	5	1.000
Non-papillary	23	10	13		11	12	
Marginal status							
Free margin	18	12	6	0.150	11	7	0.209
Not free margin	11	4	7		8	3	
Not applicable	4	1	3		2	2	
Primary tumor (T)							
Is, I, II	13	8	5	0.642	8	5	0.236
III, IV	18	8	10		8	10	
Not applicable	2	1	1		0	2	
Reginal lymph node (N) metastasis							
No	15	7	8	0.879	7	8	0.879
Yes	9	5	4		5	4	
Not applicable	9	5	4		4	5	
Distance metastasis (M)							
No	15	6	9	0.481	8	7	0.384
Yes	5	3	2		1	4	
Not applicable	13	8	5		7	6	
TNM stage							
I,II	9	7	2	0.089	5	4	0.570
III,IV	23	9	14		11	12	
Stage unknown	1	1	0		0	1	

*P<0.005, statistically significant

## Discussion

Nowadays, the curative method for CCA patients requires not only surgical treatment but also adjuvant chemotherapy to improve the overall survival of patients [4]. Although various chemotherapeutic agents have been clinically tested, a standard chemotherapy for CCA patients has not been established. Based on the ABC trial guidelines, clinical trial phase 2 studies in biliary tract cancer, gemcitabine and gemcitabine plus cisplatin have been used in clinical practice [5]. However, the individual drug response pattern of the patients concerned is the key to successful of chemotherapy [6, 20]. Therefore, a method to determine biomarkers that can predict the individual chemotherapy response is urgently required not only to screen for which drug is suitable for which patients, but also to minimize the adverse effects.

The histoculture drug response assay (HDRA) is an *in vitro* culturing method that reflects *in vivo* properties and shows a high sensitivity and specificity with respect to clinical drug response [21, 22]. The consistency of tumor tissue responses tested with chemotherapeutic agents in HDRA and the clinical drug response have been report for various types of solid tumors [8–10, 21]. In addition, the expression of proteins involved in the gemcitabine metabolism pathway, DCK, hENT-1 and RRM1, and the expression of MT and ERCC1 causing cisplatin resistance, have been reported to directly affect the gemcitabine and cisplatin responses [14–18]. This suggests that both the HDRA method and the expression of the proteins may provide information on the advantages in the clinical use of these chemotherapeutic agents for CCA patients. Therefore, this study aimed to explore the individual response pattern of CCA patients to gemcitabine, cisplatin and gemcitabine plus cisplatin using the HDRA method, as well as exploring the expression of DCK, hENT-1, RRM1, and MT and ERCC1 in the tissues of CCA patients in the same cases in a prospective study. Moreover, the relationship between the response pattern from HDRA and the expression of DCK, hENT-1, RRM1, MT and ERCC1 were analyzed.

Here, we firstly carried out HDRA on the resected tissues of CCA patients. These were tested with gemcitabine, cisplatin and gemcitabine plus cisplatin, and the cut-off of gemcitabine and cisplatin determined. The %IR were 38.46% and 43.28% for 1000 and 1500 μg/ml of gemcitabine, respectively. These were set as the cut-off for defining the tumor tissues into response and non-response groups to gemcitabine. This result corresponds to that found in previous studies in which the cut-off for gemcitabine ranged from 30%-50% in other solid tumors [9, 23, 24]. For cisplatin, %IR of 39.05% and 32.65% were set as the cut-off for 20 and 25 μg/ml of cisplatin, respectively. These results are close to the %IR of cisplatin in a previous study on esophageal cancer [23]. Our results reveal that most of the tumor tissues of CCA patients were in the non-response group to both concentrations of gemcitabine, as well as 20 μg/ml of cisplatin. On the other hand, most of the tumor tissues of CCA patients responded to 25 μg/ml of cisplatin. Moreover, we also compared the effectiveness of chemotherapies between gemcitabine and gemcitabine plus cisplatin. The results indicated that gemcitabine plus cisplatin showed a significantly higher effectiveness than gemcitabine alone. This result corresponds to the results from clinical drug response trials in both of CCA and biliary tract cancer [5, 25].

We further explored the expression of DCK, hENT-1, RRM1, MT and ERCC1 in CCA tissues from the same cases as were analysed with HDRA and determined the correlation between the expression level of each protein with the individual drug response pattern in HDRA as well as the clinicopathological data of the CCA patients. The results revealed that a low expression of hENT-1 was significantly associated with the non-response to gemcitabine tumor tissues in HDRA. This is similar to the results from the clinical drug response of biliary tract cancer. The positive expression of hENT-1 significantly correlated with stable disease and a partial clinical response [15]. Moreover, the mRNA expression of hENT-1 significantly decreased in gemcitabine-resistant CCA cell lines [26]. Additionally, the low expression of hENT-1 was also significantly associated with advance stage CCA. The association between the expression of hENT-1 and the cancer stage has also been reported in CCA, as well as in pancreatic cancer, although it did not reach significance [27, 28]. A high expression of MT and ERCC1 was significantly associated with non-response to cisplatin in HDRA. The association between the expression of MT and clinical drug response was also studied in germ cell tumors but did not reach significance [29]. However, in bladder cancer patients the overexpression of MT significantly correlated with cisplatin resistance in the clinic[30]. The expression of MT was also observed *in vitro* in cisplatin-resistant ovarian cancer cell lines when compare to cisplatin-sensitive cell lines [17]. Our results for ERCC1 concur with a study of head and neck cancer which reported that a low expression of ERCC1 was significantly associated with a complete response to cisplatin in the clinic[18]. Additionally, in gastric cancer ERCC1 mRNA expression from tumor tissue was conversely associated with cisplatin response [31]. Apart from those proteins, multidrug resistance protein (MRP) also has been report as gemcitabine and cisplatin sensitive factor. The MRP1 is related to gemcitabine resistance while MRP2 is related to cisplatin resistance. However, previous study in CCA, overexpression of MRP1 was found in gemcitabine resistance cell line when compared to parental cell. However, gemcitabine resistance cell not only resistant to gemcitabine but also other drugs such as 5-FU and doxorubicin [26]. Therefore, from this study can be concluded that role of MRP is not directly specific to gemcitabine. Moreover, the expression of MRP2 cannot be detected in CCA [32]. Therefore, the expression of MRP might not be suitable as the predictive marker for gemcitabine and cisplatin resistance in CCA.

Our study indicates that HDRA can reflect the clinical drug response and provide clinically useful information on CCA patients with respect to the chemotherapeutic agent of choice on an individual basis. In addition, the expression level of hENT-1, MT and ERCC1 may also serve as molecular predictive biomarkers for gemcitabine and cisplatin sensitivity in CCA. As the current study is prospective, the clinical chemotherapy responses of current CCA patients are still required, and the relationship between the response pattern to chemotherapy from HDRA and clinical drug response of CCA patients requires further study.

## Conclusion

We examined the individual drug response of CCA patients using HDRA and the expression level of DCK, hENT-1, RRM1, MT and ERCC1in tissues of CCA patients. This study demonstrates the effectiveness of gemcitabine, cisplatin and gemcitabine plus cisplatin in tissues of CCA patients and their association with the expression of these proteins. The association of expression of the proteins with clinicopathological data of CCA patients was also investigated and demonstrated that HDRA and protein expression may serve as a method to predict the individual drug response pattern in CCA patients.
